# Research waste before research begins: the problem of research-priority mismatch in low- and middle-income countries

**DOI:** 10.7189/jogh.16.03028

**Published:** 2026-08-28

**Authors:** Farrokh Habibzadeh

**Affiliations:** Department of Medical Journalism, Shiraz University of Medical Sciences, Shiraz, Iran

**Keywords:** research waste, research priority setting, responsible research assessment, scientometrics, low- and middle-income countries, science policy

## Abstract

Scholarship on research waste has concentrated on what goes wrong after a question has been chosen: flawed design, selective reporting, publication bias, and the slow attrition of findings that never reach the people who could use them. A more upstream loss has drawn far less scrutiny – the commitment of scarce resources to questions poorly matched to the needs of the populations expected to benefit from them. In this viewpoint, I argue that such research–priority mismatch is itself a distinct form of waste, and that its costs fall most heavily on low- and middle-income countries, where research budgets are thin and the opportunity cost of every funded project is correspondingly steep. Drawing on work in meta-research, scientometrics, responsible research assessment, and science policy, I examine how academic incentives can favour internationally visible topics over locally consequential ones, and how publication-based promotion, journal prestige, and bibliometric indicators can quietly entrench that preference. To name priority mismatch as waste is not to reject international collaboration or curiosity-driven inquiry; it is to ask for proportion between global participation and local relevance. I close with a review of ongoing reforms in funding, evaluation, and academic reward that might draw research investment back toward public benefit.

For more than a decade, research waste has held a conspicuous place in debates about how science is funded and judged. A now-substantial body of work has shown that a large share of research investment never yields its full value because studies pose questions of little importance, lean on inadequate methods, go unpublished, or are reported so poorly that others cannot build on them [1–3]. These findings have spurred welcome reforms in transparency, reproducibility, and methodological rigour.

Yet one form of inefficiency has kept to the margins of the conversation. A study can be methodologically sound, ethically conducted, duly published, and widely cited, and still add little to the welfare of the society that paid for it. The unease this provokes points to a question older than any metric: should research be judged only by its quality and its visibility, or also by its fit to the problems that press most urgently on the people who fund it?

The question gains force wherever resources are scarce. There, every allocation carries an opportunity cost; money committed to one question is money withheld from another. When scarce funds are spent year after year on problems of limited local consequence while urgent needs go unstudied, the cumulative loss is not trivial. The result need not be bad science; it may be good science pointed in the wrong direction.

In this viewpoint, I develop the case that the research–priority mismatch is a neglected source of research waste, and consider how academic incentives, bibliometric evaluation, and international research structures can contribute to it.

## THE FIRST STAGE OF WASTE

The modern discussion of research waste is usually traced back to Chalmers and Glasziou [1], who estimated that a large fraction of biomedical investment is avoidably squandered and located the losses across four sequential stages spanning from the choice of question to the reporting of results. The argument was later elaborated into a coordinated programme of reform encompassing design, regulation, accessibility, and reporting [2,4], and then revisited to ask, pointedly, who had been listening [3]. A decade on, its authors concluded that the waste remained, in their word, a ‘scandal’ [5].

Much of this work has trained its attention on methodological efficiency: prospective registration, transparent reporting, data sharing, and the disciplined synthesis of what is already known. The gains have been real. However, waste can occur before any of these considerations apply. The choice of question is itself an act of allocation, and the first of the four stages identified at the outset concerns precisely that – the setting of priorities [6]. If the chosen question addresses a matter of marginal significance while more pressing needs are left untouched, resources are consumed without commensurate public return. In the parlance of economics, the problem is opportunity cost; in policy, priority setting; and in ethics, stewardship.

This is not a rival account of research waste so much as a complement to it. Efficiency and relevance are separate dimensions of value. A trial can be impeccably designed, yet trained on a question few need answered; a finding can be statistically secure, yet as Ioannidis warned of much of the published record, fragile or beside the point [7]. To recognise priority mismatch as waste is, therefore, to widen the inquiry from how research is done to why a given line of research is undertaken at all.

## WHERE SCARCITY SHARPENS THE STAKES

Every research system must choose among priorities, but the consequences of choosing badly are not evenly distributed. Wealthier countries can often sustain both nationally relevant work and large portfolios of curiosity-driven inquiry. Resource-constrained systems rarely enjoy that latitude.

Many low- and middle-income countries devote only a small share of national income to research and development while contending with heavy burdens in health, education, environment, and economic development. The misalignment between the research the world needs and the research it actually performs is long-standing and well documented: most health research and development continues to address conditions that account for a minority of the global disease burden, a gap first quantified more than three decades ago and only partly narrowed since [8,9]. This responsiveness is uneven and incomplete even where funders are broadly responsive to burden [10]. A recent analysis linking millions of publications to two decades of burden data found that research effort and need have drifted apart, and projected that the divergence would widen – a trajectory that the retreat of major public funders threatens to accelerate [11]. Against this backdrop, inefficient allocation in a poorly resourced system does disproportionate harm.

None of this argues for a narrowly utilitarian science. Foundational discoveries routinely grow from questions whose practical worth is, at first, invisible; progress depends on intellectual range and on the patience to follow a thread without knowing where it leads. Curiosity-driven inquiry and societally directed research should not be understood as competing claims on scientific legitimacy, but as complementary elements of a healthy research system. The claim here is narrower and harder to refuse: when resources are scarce, the persistent neglect of society’s gravest problems becomes difficult to defend. Research systems carry a dual obligation: to enlarge the common store of knowledge and to attend to problems of national consequence. The balance between the two deserves to be chosen deliberately, rather than inherited by default.

## THE GRAVITY OF INCENTIVES

Research agendas do not assemble themselves. They take shape under incentive systems that act on researchers, institutions, journals, and funders alike. Appointment and promotion still turn heavily on publication counts, journal prestige, citation tallies, and the *h*-index and its many descendants [12,13]. Investigators respond to these signals rationally, and early-career scientists, with the most to lose, respond most keenly of all.

The behaviour should not be read as personal failing. Researchers work within systems they did not build; where reward attaches to measurable output, effort flows towards what can be measured. The difficulty arises when the measures reward visibility more reliably than relevance. The mechanism is the one Strathern, glossing Goodhart, set out for the audited university: once a measure becomes a target, it ceases to be a good measure [14]. A question likely to attract citations can come to look more attractive than a question likely to change lives, though the two are seldom the same. As critics of the journal impact factor have argued for almost thirty years, what drives scholarly visibility is not necessarily what drives practical use [15].

Used with care, bibliometric indicators can inform judgement; used in isolation, they distort it. The San Francisco Declaration on Research Assessment and the Leiden Manifesto both arose in answer to exactly this overreach, urging that quantitative measures support expert judgement, rather than supplant it [12,16]. The deeper trouble is that visibility and usefulness are read by different instruments. A locally grounded intervention study may transform public health practice while gathering modest citations; a narrow specialty topic may accumulate citations while touching ordinary life hardly at all. Neither outcome is troubling in itself. Trouble begins when evaluation rewards citation visibility while overlooking societal value – when the latter, harder to count and slower to appear, goes unweighed for want of a convenient number [17]. Over time, this quiet pull can reshape a national agenda without anyone having decided that it should.

## A WIDER SKY: GLOBALISATION AND THE LOCAL

Contemporary science is, by temperament and by infrastructure, international. Researchers collaborate across borders, share data, and publish in journals read on every continent. The benefits of this openness are immense. Yet the same currents can tilt the hierarchy of what counts as important. High-impact journals serve broad international readerships, and topics framed as globally salient may draw more editorial interest than questions whose significance is chiefly regional or national. The pattern is understandable and largely no one’s fault; its cumulative effect is that some locally vital problems remain less visible in the international literature than their importance warrants.

Nor does international orientation necessarily imply diminished local value. Work directed at globally salient questions may generate methods, infrastructure, expertise, and collaborations that ultimately benefit domestic research systems, while discoveries made elsewhere often prove relevant far beyond the settings in which they originated. The relationship between global engagement and local relevance is, therefore, not a zero-sum one. The concern is narrower: when the pursuit of international visibility becomes a dominant criterion of success, research agendas may drift away from problems whose importance is substantial locally but less apparent internationally. The issue is not participation in global science, but the absence of sufficient counterweights to ensure that locally consequential questions remain part of the research portfolio.

There is a structural dimension as well. In what Marginson and Xu describe as a centre-periphery system [18], the agenda set at the centre is not always consistent with the needs of the periphery, and scholars trained in dominant settings may carry imported interests into systems whose problems differ. Such exchange can enrich scientific communities, yet it can also quietly seed priority mismatch when borrowed agendas are adopted without adaptation. The concern is sharpened by a now-vigorous debate on the coloniality of global health. Abimbola has termed this dynamic the ‘foreign gaze’, referring to the tendency for research in one setting to be shaped by the perspectives, priorities and expectations of researchers and institutions from elsewhere, particularly those with greater control over funding and agendas [19,20]. The question is not whether international priorities are legitimate – they often are – but whether local priorities retain enough room within national portfolios to be pursued at all.

## STEWARDSHIP AND THE ETHICS OF DIRECTION

Research funding is a social investment. Whether the money comes from governments, charities, universities, or, in the end, taxpayers, decisions about where it goes carry an ethical charge [21,22]. The ethics of priority setting is most often discussed under the headings of fairness, equity, and inclusion. Less often examined is the possibility that the steady neglect of a community’s most serious needs is itself a kind of distributive injustice. People who contribute to the cost of research may reasonably expect that at least part of the resulting effort will bear on the conditions that shape their lives.

That expectation does not require every project to demonstrate immediate practical impact; it asks only that a research portfolio maintain a defensible relationship to the priorities of those it serves. Where the mismatch becomes severe, questions of accountability are unavoidable. Seen this way, the research–priority mismatch is not merely a technical inefficiency to be optimised away; it is a matter of stewardship – of what we owe to the publics whose resources, and whose futures, are at stake [23].

## TOWARD A BROADER EXCELLENCE – AND HOW TO REACH IT

The language of excellence sits at the centre of research policy, yet the word itself remains contested. In practice, excellence is too often inferred from publication and citation performance alone. Those signals capture something of scientific influence, but they do not exhaust the contributions research can make. A fuller conception would hold several dimensions of value in view: methodological rigour and originality, international visibility, societal relevance, implementation potential, policy influence, and contribution to national priorities. This is the direction in which the reform of research assessment has been moving – from the Leiden Manifesto and the Declaration on Research Assessment to the Metric Tide, the Hong Kong Principles, and most recently, the Coalition for Advancing Research Assessment [12,16,24–26]. Quantitative indicators need not be discarded; they need to be placed within a broader, judgement-led frame.

If the research–priority mismatch is accepted as a form of waste, practical consequences follow. Funders should strengthen the machinery for identifying and periodically refreshing national research priorities through transparent, inclusive exercises involving researchers, policymakers, practitioners, and the communities affected; well-developed checklists already exist to guide such work [6,27]. Universities should broaden the criteria for advancement so that a strong publication record is complemented by evidence of societal contribution, policy engagement, implementation, and capacity building [25,26]. Journals can help by treating rigorous work on regional and national problems as the serious science it is, rather than as parochial. Research assessment systems should pair quantitative measures with informed qualitative judgement, never allowing the metric to stand in for the assessment it was meant to assist [12]. Researchers can ask how their work relates to the needs of the communities they serve – a habit of reflection that need not constrain curiosity so much as give it somewhere worth going.

These changes need not prescribe what every researcher should study. Their purpose is more modest: to ensure that relevance has a place alongside visibility when scarce resources and research careers are being directed. A healthy research system should leave room for the unexpected while remaining answerable to the important.

## CONCLUSIONS

Research waste is usually pictured as flawed methods, poor reporting, and findings that fail to circulate. Those concerns remain real and pressing. However, an exclusive focus on methodological efficiency risks passing over a more fundamental question: are scarce research resources being aimed at the problems that matter most? The research–priority mismatch deserves to be counted as a distinct source of waste, above all where resources are limited and societal needs are large. The argument is not a quarrel with international science, with curiosity-driven inquiry, or with excellence properly understood; it is a request to widen what we mean by value.

Science casts light into the dark; the direction in which the beam is turned is a choice, and one that matters. A system that rewards visibility while neglecting relevance risks producing knowledge that is impressive yet insufficiently useful. Aligning research priorities with societal needs will not abolish waste. It may, however, help ensure that the dividends of scientific effort reach beyond publications and indicators to the communities whose futures depend on them.

## Data Availability

**Data availability:** No new data were generated or analysed in the preparation of this viewpoint.
